# The antioxidants resveratrol and *N*-acetylcysteine enhance anthelmintic activity of praziquantel and artesunate against *Schistosoma mansoni*

**DOI:** 10.1186/s13071-019-3566-9

**Published:** 2019-06-20

**Authors:** Maria João Gouveia, Paul J. Brindley, Carlos Azevedo, Fátima Gärtner, José M. C. da Costa, Nuno Vale

**Affiliations:** 1 Center for the Study of Animal Science, CECA-ICETA, University of Porto, Praça Gomes Teixeira, Apartado 55142, 4051-401 Porto, Portugal; 2 i3S, Instituto de Investigação e Inovação em Saúde, Universidade do Porto, Rua Alfredo Allen, 208, 4200-135 Porto, Portugal; 3 ICBAS-UP, Institute of Biomedical Sciences Abel Salazar, University of Porto, Rua de Jorge Viterbo Ferreira, 228, 4050-343 Porto, Portugal; 4 Department of Microbiology, Immunology and Tropical Medicine and Research Center for Neglected Diseases of Poverty, School of Medicine and Health Sciences, George Washington University, 20037 Washington, DC USA; 5 Laboratory of Cell Biology, Institute of Biomedical Sciences (ICBAS/UP), University of Porto, Rua Jorge Viterbo Ferreira, 228, 4050-313 Porto, Portugal; 6 Institute of Molecular Pathology and Immunology of the University of Porto (IPATIMUP), Rua Júlio Amaral de Carvalho, 45, 4200-135 Porto, Portugal; 7 National Health Institute Dr. Ricardo Jorge (INSA), Rua Alexandre Herculano, 321, 4000-055 Porto, Portugal; 8 Laboratory of Pharmacology, Department of Drug Sciences, Faculty of Pharmacy, University of Porto, Rua de Jorge Viterbo Ferreira 228, 4050-313 Porto, Portugal

**Keywords:** Anthelmintic, Resveratrol, *N*-acetylcysteine, Biomolecules, Schistosomula, *Schistosoma mansoni*

## Abstract

**Background:**

Treatment of schistosomiasis has relied on the anthelmintic drug praziquantel (PZQ) for more than a generation. Despite its celebrated performance for treatment and control of schistosomiasis and other platyhelminth infections, praziquantel has some shortcomings and the inability of this drug to counteract disease sequelae prompts the need for novel therapeutic strategies.

**Methods:**

Using a host-parasite model involving *Biomphalaria glabrata* and *Schistosoma mansoni* we established mechanical transformation of *S. mansoni* cercariae into newly transformed schistosomula (NTS) and characterized optimal culture conditions. Thereafter, we investigated the antischistosomal activity and ability of the antioxidants *N*-acetylcysteine (NAC) and resveratrol (RESV) to augment the performance of praziquantel and/or artesunate (AS) against larval stages of the parasite. Drug effects were evaluated by using an automated microscopical system to study live and fixed parasites and by transmission electron microscopy (TEM).

**Results:**

Transformation rates of cercariae to schistosomula reached ~ 70% when the manipulation process was optimized. Several culture media were tested, with M199 supplemented with HEPES found to be suitable for *S. mansoni* NTS. Among the antioxidants studied, RESV alone or combined with anthelminthic drugs achieved better results rather *N*-acetylcysteine (NAC). TEM observations demonstrated that the combination of AS + RESV induced severe, extensive alterations to the tegument and subtegument of NTS when compared to the constituent compounds alone. Two anthelmintic–antioxidant combinations, praziquantel-resveratrol [combination index (CI) = 0.74] and artesunate-resveratrol (CI = 0.34) displayed moderate and strong synergy, respectively.

**Conclusions:**

The use of viability markers including staining with propidium iodide increased the accuracy of drug screening assays against *S. mansoni* NTS. The synergies observed might be the consequence of increased action by RESV on targets of AS and PZQ and/or they may act through concomitantly on discrete targets to enhance overall antischistosomal action. Combinations of active agents, preferably with discrete modes of action including activity against developmental stages and/or the potential to ameliorate infection-associated pathology, might be pursued in order to identify novel therapeutic interventions.

**Electronic supplementary material:**

The online version of this article (10.1186/s13071-019-3566-9) contains supplementary material, which is available to authorized users.

## Background

Schistosomiasis is considered the most important helminthic disease of humanity in terms of morbidity and mortality; more than 240 million people are infected with schistosomes, and another 700 million are at risk of infection [1–4]. Despite the fact that control strategies have been employed to block transmission and reduce the disease burden, including mass and targeted chemotherapy, improvements to sanitation and modification of the environment, and the use of molluscides [5], schistosomiasis remains a major public health problem in sub-Saharan Africa [3, 6]. Historically considered restricted to the tropics and subtropics where suitable intermediate host snails also are endemic, transmission of schistosomiasis has recently re-emerged in southern Europe [7]. Currently, chemotherapy is the first-line tool to minimize the prevalence and incidence of schistosomiasis [8–10]. Indeed, for the past 40 years praziquantel (PZQ) has been recommended by the World Health Organization for the treatment of all forms of schistosomiasis [11]. PZQ is inexpensive, given by mouth, readily available and well-tolerated, and hence suitable for mass drug treatment campaigns [11]. However, whereas PZQ is active against the adult developmental stages and against young schistosomula within a day or so of infection, it displays poor efficacy against schistosome eggs and the developing and migrating immature schistosomula and young adult forms [12]. This likely explains and contributes to low cure rates and rapid re-infection where residents of endemic sites are frequently infected with both juvenile and adult parasites concurrently. For effective treatment and sustainable control, PZQ retreatment must be maintained on a regular basis.

On the other hand, the dependence on PZQ raises legitimate concerns about the appearance of drug resistance [12–14]. Although widespread resistance has not been convincingly demonstrated, field and experimental isolates displaying reduced sensitivity to PZQ have been described from several countries [15]. The discovery and development of novel effective drugs, and also new drug targets, has been considered a research priority. PZQ derivatives do not improve antischistosomal activity. Frequently, the promising *in vitro* activity of a candidate PZQ-derivative has not been translated into antischistosomal activity *in vivo* [10]. There is a reawakening of the need and value to search for alternative chemotherapeutic tactics, such as combinations of drugs and drug repurposing [16–18].

PZQ alone cannot capably reverse pathological sequelae of schistosomiasis [19], and new therapies against schistosomiasis should focus also on this problem in addition to anthelmintic performance. During the past 30 years, artemisinin derivatives, such as artesunate (AS), have been shown to have antischistosomal activity both *in vitro* and in animal models. In marked contrast to PZQ, AS exhibits potent activity against juveniles whereas the invasive stages and adult worm are less susceptible. Moreover, adult female worms are somewhat more susceptible than the males. Although the mechanism of action of AS against schistosomes is not well understood, the glycogen content of worms is reduced by a reduction in glucose uptake, an increase in glycogen phosphorylase activity, and by inhibition of enzymes involved in glucose metabolism [20, 21].

During schistosomiasis, alterations occur in organs and tissues including disturbance to cellular antioxidant systems, which likely degrade the detoxification process of exogenous or endogenous free radical liberation, e.g. reactive oxygen species (ROS), which originate during the immunological response [22, 23]. On the other hand, reactive electrophilic compounds, e.g. estrogen-like metabolites, capable of reaction with DNA to form DNA-adducts and liberation of ROS, have been implicated as initiators of squamous cell carcinoma during urogenital schistosomiasis caused by infection with *Schistosoma haematobium* [24]. In this situation, the need for antioxidants increases to counteract reactive xenobiotics arising from oxidation [25–27], and in support of immunological and inflammatory responses directed at schistosome eggs in tissues [26]. Moreover, antioxidants might prevent DNA damage [28] and block the initiation of carcinogenesis [29].

Antioxidants such as *N*-acetylcysteine (NAC) and resveratrol (RESV) might ameliorate hepatic redox homeostasis during schistosomiasis. In addition, their protective effects against liver fibrosis induced by granuloma formation during infection may account partially for the ability of these antioxidants to inhibit or ameliorate the formation of schistosomal toxic products and render their impact reversible [26, 30]. NAC is an acetyl derivative of l-cysteine containing a thiol group, which participates in known biochemical pathways including its role as a precursor of cysteine, which is the rate-limiting component of glutathione (GSH). Moreover, NAC itself serves as an antioxidant by reacting directly with free radicals [31]. RESV is a 3,4,5-trihydroxylstilbene, a naturally occurring polyphenol occurs in flowering plants where it plays a role in homeostasis during environmental stress [32]. RESV exhibits neuroprotective and cardio-protective benefits [33, 34]. Not only is RESV an antioxidant, it also induces others intracellular antioxidant activities [34].

It has been emphasized that more research should be undertaken to investigate whether combinations with active compounds would reveal synergistic effects that could contribute to enhanced anthelmintic outcomes [35]. Despite having the above-described positive attributes, both and PZQ and AS present some drawbacks. We speculated that combinations of these anthelmintic drugs with antioxidant biomolecules might enhance the antischistosomal performance of PZQ and AS. We investigated the schistosomicidal activity *in vitro* of combinations of PZQ and AS with the antioxidants NAC and RESV against newly transformed schistosomula (NTS) of *S. mansoni*.

## Methods

### Drugs and media

PZQ, NAC, Medium 199, HEPES (4-(2-hydroxyethyl)-1-piperazine ethane sulfonic acid) (1M), l-glutamine, penicillin and streptomycin, Hank’s balanced salt solution (HBSS), and amphotericin B were purchased from Sigma-Aldrich (Lisboa, Portugal); heat inactivated fetal bovine serum (iFBS) from Lonza (Basel, Switzerland); RESV from Santa Cruz Biotechnology (Dallas, TX, USA); and AS from Bertin Pharma (Montigny-le-Bretonneux, France). For *in vitro* assays, stock solutions of test compounds (2–4 mg/ml) were prepared in 100% dimethylsulfoxide (DMSO; Sigma-Aldrich) and stored at 4 °C.

### Transformation of cercariae into newly transformed schistosomula (NTS)

To evaluate the schistosomicidal activity of antioxidants and whether or not they might augment the activity of anthelmintic drugs, cercariae of *S. mansoni* were mechanically transformed into schistosomula by vortexing transformation. NTS were obtained by mechanical transformation of *S. mansoni* cercariae shed from *Biomphalaria glabrata* for 2–3 h after exposure to light, with mechanical transformation performed as described [36]. Some parameters including duration of cercarial suspension on ice (30 to 60 min), centrifugation time (5 to 10 min), centrifugal force (800 to 1000× *rpm*) and steps during purification (3 to 5) were modified to enhance conversion rates of cercariae into schistosomula. The final step consisted of chilling the cercarial suspension on ice for 60 min, after which cercariae were pelleted by centrifugation at 1000× *rpm* at 4 °C for 10 min. The cercarial pellet was resuspended in 2 ml of cold HBSS containing 2% amphotericin B, mixed vigorously through a pipette, vortexed for 4 min to induce tail shedding, and incubated on ice for 10 min to concentrate the NTS. The tail-rich supernatant was decanted and discarded, and the pelleted schistosomula re-suspended in 7 ml of cold HBSS, with this step repeated five times. The conversion rate was calculated by counting the total number of cercariae before transformation in relation to the total number of schistosomula obtained after purification (Table 1).

**Table 1:** Conversion rates for newly transformed schistosomula (NTS) *of Schistosoma mansoni* obtained using a modified vortex transformation procedure

Experiment	Description	Cercarial suspension (ml)	No. of cercariae	No. of schistosomula	Conversion rate (%)	Mean ± SD	Observations
1	Reduced motility: 30 min on ice; centrifugation: 800× *rpm*, 5 min, 4 °C; purification: 3 steps (15 min on ice)	35	3500	1750	50.0	^a^	Experiment 1 and 2: lower conversion rate; considerable number of tails and cercariae detected
2	Reduced motility: 30 min on ice; centrifugation: 800× *rpm*, 5 min, 4 °C; purification: 5 steps (15 min on ice)	38	6080	2800	47.0	61.4 ± 10.2	
3		22.5	3825	2900	75.8		Despite an increase in the schistosomula conversion rate, several tails were observed in wells
4	Reduced time between purification steps (15–10 min) (centrifuged 2× in order to obtain a solid pellet); slight increase of vortexing time	45	5850	3240	55.0	^a^	Alteration of culture supplemented media M199, 20 mM HEPES, 10% iFBS. It is probable that the time of initial suspension on ice was insufficient to decrease parasite motility, which led to a loss of cercariae after supernatant removal
5		25	10,000	–	–		Due to higher number of cercariae in initial suspension, it is probable that the time on ice was not enough to reduce motility. Consequently, it did not form a solid pellet after centrifugation leading to release of cercariae after removal of supernatant
6	Experiments 6–12: reduced motility: 60 min on ice; centrifugation: 1000× *rpm*, 10 min, 4 °C; purification similar to experiments 4 and 5; introducing checkpoints during the transformation method	50	5250	3720	70.9	68.6 ± 4.8	Fewer cercariae and tails detected. Good parasite fitness. Increased of concentration of iFBS to 15%. Final culture media established as supplemented M199 with HEPES and 10% FBS
7		100	11,750	7380	62.8		Due to a great number of cercariae, mechanical transformation was performed on separated tubes
8		40	12,000	8640	72.0		Experiment 8 and 9: lower number of tails and cercariae. Good parasite fitness
9		50	3000	2250	75.0		
10		27.5	1640	1040	63.4		Lower number of cercariae because several infected snails had died, thus only a few snails were infecting and shedding cercariae. In this case, we only performed 4 steps for purification in order to reduce loss of schistosomula during the process
11		50	4625	3000	64.8		Lower number of tails and cercariae, as well good parasite fitness
12		50	7375	5265	71.4		

^a^Unable to calculate mean and standard deviation (SD)

### Optimizing the culture conditions

After transformation of cercariae into NTS, optimal culture conditions were established iteratively. Due to microbial contamination from the schistosome-infected snails and snail excrement, culture media for NTS were supplemented with 100 U/ml penicillin and 100 μg/ml streptomycin [37]. NTS were incubated in supplemented media M199 at 37 °C in 5% CO_2_ in air [37]. NTS were placed in 96-well flat-bottom plates (Nunclon, Roskilde, Denmark) and incubated with M199 supplemented with 20 mM HEPES or 7.5% sodium bicarbonate and increasing concentrations of heat inactivated fetal bovine serum (iFBS), 5–15%, 37 °C in 5% CO_2_ in air. The viability of NTS, at 50–100 larvae per well, was assessed daily based on morphology and motility using a semi-quantitative grading, where a score of 3 indicated normal activity without morphological changes; 2 indicated activity with some morphological changes and/or granularity; 1 indicated minimal activity, severe morphological changes and granularity; and 0 indicated no movement seen, severe granularity, non-viable [37–39] as observed under bright field at 10–40× magnification with an inverted microscope (Nikon Phase Contrast 2, LDW 0.52; Nikon, Tokyo, Japan). Schistosomula were considered to have died when movement was not evident after 90 s [38]. Micrographs were captured using a camera (PowerShot A360; Canon, Maryland, USA).

### Antischistosomal activity

A concentration of 50–100 NTS per 100 μl in pre-heated (37 °C) optimal culture media was placed in 96-well flat-bottom plates (Nunclon) and incubated for 24 h at 37 °C in 5% CO_2_ in air [38]. Culture media, 250 μl final volume per well, were supplemented with test compounds at increasing concentrations. NTS incubated in medium containing the maximum DMSO concentration, 2% v/v, served as the vehicle control. In a first screening, NTS were incubated for 72 h at the highest concentration (100 μM) of PZQ, AS, NAC and RESV alone and combined (e.g. PZQ-NAC) 1:1 (v/v). Secondary screening was performed iteratively based on antioxidant concentration performance during the initial screen. At the second screen, serial dilutions from 10 to 100 μM were tested. Initially, viability and morphological alterations 1, 17, 24 and 48 h post-exposure were assessed using inverted microscopy. After 72 h, NTS viability was assessed with the assistance of automated microscopy (LionHeart FX, BioTek, Winooski, VT, USA) fitted with Gen5 v.3.0 software to process and analyze data, to capture color bright field and fluorescence Texas Red channel (586 nm) images. Propidium iodide [PI; 0.5 mg/ml in sodium citrate (1%)] was added to each well and NTS incubated for 15 min a 37 °C [40, 41]. The principle is based on the different membrane permeability to the membrane-impermeable fluorescent DNA intercalating agent PI which stains membrane-compromised cells (red fluorescence) [40]. The performance of each combination was characterized using a combination index (CI), where CI > 0.1 indicates very strong synergism, CI: 0.1–0.3 strong synergism, CI: 0.3–0.7 synergism, CI: 0.7–0.85 moderate synergism, CI: 0.85–0.9 slight synergism, CI: 0.9–1.1 nearly additive and CI > 1.1 antagonist, as previously described [42, 43]. This was calculated with CompuSyn v.1.0 (ComboSyn, Inc., Paramus, NJ, USA). Each concentration of anthelmintic drug, antioxidant alone and in combination was tested in duplicate; the assays were performed at least twice.

### Transmission electron microscopy (TEM)

For evaluation of ultrastructural alterations induced by AS, RESV or AS + RESV (1:1) at 100 μM and post-exposure of 72 h, the NTS treated and untreated (controls) were fixed with 2.5% glutaraldehyde in 0.2 M sodium cacodylate, pH 7.4 for 4 h. After fixation, the worms were washed overnight in the same buffer and post-fixed in 1% osmium tetraoxide (OsO_4_). Subsequently, fixed NTS were dehydrated in an ascending, graded ethanol series and embedded in Epon epoxy resin. Semi-thin sections were stained with methylene blue-Azur II. Ultrathin sections were double-contrasted with aqueous uranyl acetate and lead citrate. Ultrastructural features of NTS in the sections were observed and images documented with TEM using a JEOL 100CXII microscope (JEOL, Massachusetts, USA) operated at 60 kV and equipped with a Gatan digital camera (Gatan, California, USA).

## Results

### Mechanical transformation

The mechanical transformation of cercariae of *S. mansoni* into NTS using the revised protocol yielded an average conversion rate of 68.6 ± 4.8% (mean ± SE) compared to 47% using an earlier method [36], as well as fewer cercarial tails in the culture plates during the downstream assays (Table 1, 12 experiments). Low conversion rates seen in Experiments 1 and 2 likely were related to the shorter time on ice; hence, the larvae were more mobile. Centrifugation at 800× *rpm* for 5 min might be insufficient to pellet the larvae, so that cercariae may be inadvertently discarded with the supernatant. During Experiment 3, the conversion rate was higher than in Experiments 2 and 4 which may be related to the lower number of cercariae in the initial suspension. Thus, the time on ice might be sufficient to reduce motility and/or the reduction of the volume of initial suspension may favor a fast sedimentation and formation of solid pellet of cercariae after centrifugation. During the mechanical transformation, it is necessary to consider additional parameters that may vary from assay to assay, including numbers of infected snails, numbers of cercariae, and volume of cercarial suspension. We observed that steps needed to be adjusted during transformation in order to improve the conversion rates. For example, if the initial number of cercariae in suspension was higher (~ 10,000 per 50 ml), more time on ice to reduce parasite motility and more vortexing time to induce removal of the tail were both required. However, it is necessary to caution that increased vortexing can injure the larvae [37]. Several checkpoints were performed in order to assess parasite fitness and tail-loss during transformation. It should be noted that the number of cercariae obtained in initial suspensions was variable (Table 1) since it varied between snails, and was dependent upon additional factors including number of infected snails and number of times the cercariae had been shed from the snails.

### Optimal culture conditions for *S. mansoni* NTS

NTS cultured in medium M199, supplemented with 7.5% sodium bicarbonate and 5% iFBS, died after 72 h. Parasites incubated in M199 supplemented with 20 mM HEPES and 10% iFBS, remained viable for at least 96 h without membrane disruption and/or marked morphological changes (Additional file 1: Figure S1). These NTS displayed an average viability value of about 2.5 (not shown). Increasing the concentration of iFBS to 15% did not enhance viability. Therefore, M199 supplemented with 20 mM HEPES and 10% iFBS appeared to be suitable for incubation of NTS by vortex transformation and was used for the drug sensitivity assays.

### *In vitro S. mansoni* NTS drug sensitivity assay

**Fig. 1:**
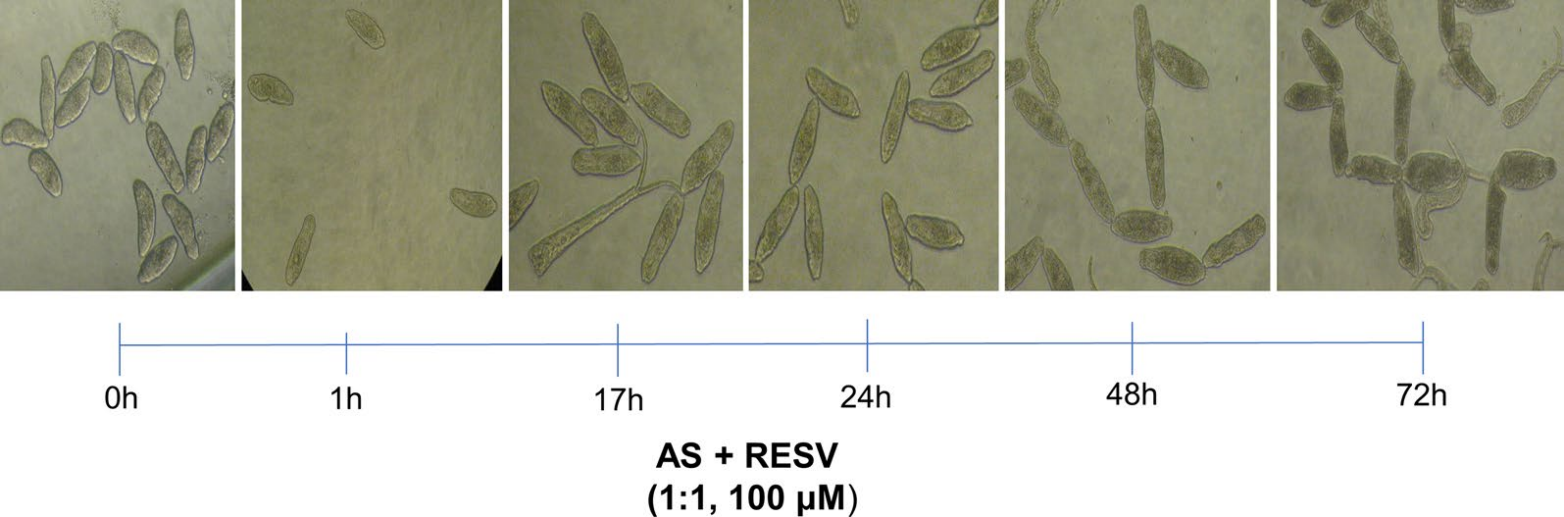
Temporal effect of exposure of schistosomula of *Schistosoma mansoni* for 72 h to anthelmintic drugs and antioxidants (drugs AS + RESV 1:1, 100 μM)

Antischistosomal activity of antioxidants (NAC and RESV) and their ability to enhance antiparasitic activity of anthelmintic drugs (PZQ and AS) was assessed on NTS derived from successful mechanical transformation. As previously described [38], in the absence of test compounds, NTS showed normal viability without any morphological changes for up to 24 h. Mild changes in granularity and motility were apparent following 48 h of incubation. NTS remained viable for at least 96 h [38]. Generally, incubation in PZQ and AS at the highest concentration (100 µM) caused severe deformity and granularity scores of 1 (slow activity and severe granulation) on the viability scale of NTS (Additional file 1: Figures S2 and S3). However, none of the drugs alone were capable of inducing the death of all NTS (Additional file 1: Table S1). Following 24 h in AS, NTS did not show significant morphological alterations in comparison to controls. However, after 48 h the larvae were granular in appearance, some markedly, irregular in shape, and movement was reduced. By 72 h, most NTS were dead (Additional file 1: Table S1). PZQ strongly decreased viability but did not cause the death of NTS. Initially, PZQ elicited an overactive phenotype progressing to overactive/degenerate but motile, i.e. NTS were motile yet severely disrupted in morphology. In addition, we observed spheroid-shaped worms (rather than vermiform), which were markedly granulated, as described by other investigators [44, 45]. Although PZQ inflicted more damage, AS was more active than PZQ inducing more mortality among NTS (Additional file 1: Table S1), consistent with other reports that indicated that AS was more effective against larval and young stages of *S. mansoni* [20]. The antioxidants NAC and RESV were tested at up to 100 µM for anthelmintic activity. Neither NAC nor RESV killed NTS after 72 h of incubation (Additional file 1: Figure S2). Nevertheless, the percentage of dead NTS induced by the Resv compound was higher rather than NAC (Additional file 1: Table S1). NAC induced only slight morphological alterations, specifically an increase in granularity at the highest concentration tested (Additional file 1: Figure S3). RESV induced an increase in granularity, and reduced motility (Additional file 1: Figure S3). To investigate synergism between PZQ or AS and the antioxidants, NTS were incubated with a constant dose ratio (1:1) of the highest concentration (100 μM) of a combination of each anthelmintic and antioxidant (Additional file 1: Table S1). Combinations of PZQ or AS with NAC achieved the same viability score and similar percentage of death as the anthelmintic drug alone (Additional file 1: Figures S2, S3 and Table S1). The morphological alterations and mortality of NTS incubated with PZQ + NAC were generally identical with those induced by drug alone (Additional file 1: Table S1 and Figure S5). The NTS exhibited less granularity and rounded shape than seen in larvae incubated in PZQ alone. Similarly, incubation with AS + NAC led to severe granularity, loss of motility and changes in shape identical with changes induced by AS alone (Additional file 1: Figures S2 and S3). Curiously, the number of dead NTS was lower in combination in comparison to the drug alone (Additional file 1: Table S1 and Figure S5). In contrast, combinations of PZQ or AS with RESV markedly enhanced the *in vitro* effects as compared with the anthelmintic alone. Indeed, the percentage of effects achieved by combinations was higher in comparison to compounds alone (Additional file 1: Table S1). Notably, only AS + RESV killed all NTS after 72 h, indicating that RESV enhanced the anthelmintic performance of AS (Additional file 1: Table S1 and Figure S5). Figure 1 depicts the temporal effect of AS + RESV at constant dose ratio (1:1) at the highest concentration used. Notably, RESV achieved better results than NAC in all combinations examined (data not shown).

**Fig. 2:**
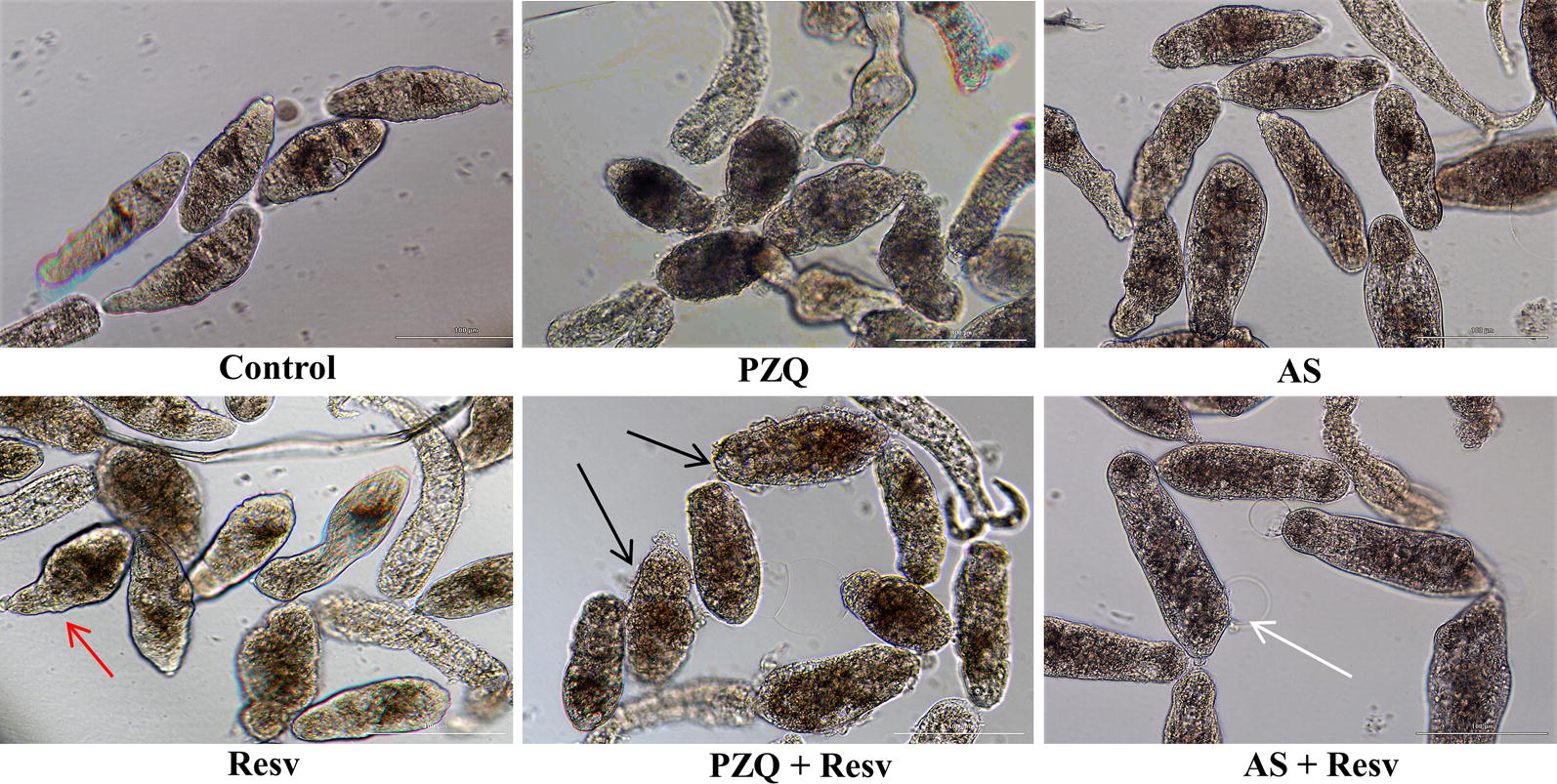
Morphological alterations manifested by schistosomula of *S. mansoni* following exposure to anthelmintic drugs and RESV and their combinations. Newly transformed schistosomula were exposed for 72 h to PZQ, AS, RESV, PZQ + RESV and AS + RESV in a dose ratio of 1:1 at highest concentration (100 μM) and compared to controls. Generally, NTS showed dark granularity and alterations in shape that were more pronounced after exposure to combinations of anthelmintics and antioxidants that to anthelmintics or antioxidants alone. PZQ induced a round/oval shaped phenotype and severely disrupted morphology. Although RESV induced some morphological alterations, the tegmental integrity of NTS larvae remained intact (red arrow). PZQ + RESV induced not only severe granularity but also blebbing (black arrows). With AS + RESV, NTS showed membrane disruption (white arrow) followed by death. Images were captured using a BioTek LionHeart FX Automated Live Cell microscope (magnification of 20×)

Figure 2 illustrates the alterations in morphology induced by the anthelmintic drugs PZQ or AS and RESV alone as well as their combination at the highest concentration, after incubation for 72 h. Real time morphological alterations of the schistosomula were assessed with an inverted microscope and with the Biotek LionHeart FX automated imaging microscopy platform. Images demonstrating relevant differences among the morphology of NTS incubated with AS, PZQ and RESV, alone and in combinations, were captured. Despite several morphological alterations induced by 100 µM RESV, the NTS maintained membrane integrity (red arrow) and showed minimal activity while PZQ induced the rounded shape effect and severe granularity, although the NTS remained motile, as previously described [44, 45]. The combination of PZQ + RESV induced significant morphological alterations, notably blebbing (black arrow). In the assays with AS, the anthelmintic alone or the combination AS + RESV induced severe granularity, alterations in shape and inactivated the NTS. Note that NTS incubated with AS + RESV suffered membrane disruption (white arrow), indicating the death of the schistosomulum.

Since RESV at maximum concentration (100 μM) showed antischistosomal effects alone and also potentiated the effects of the anthelmintic, combinations with lower concentrations of RESV were tested in the same (1:1) or in different concentration ratios in a secondary screen (Table 2). Combinations at a constant ratio (1:1) at different concentrations (10–100 μM) achieved better activity than the other constant ratios tested. As depicted in Table 2, all combinations induced higher NTS mortality compared to compounds alone (see also Additional file 1: Figure S5). At lower concentrations (< 100 μM) of anthelmintic and RESV (alone) or combined, only low to moderate activity was seen (Table 2). None killed all the NTS by 72 h and morphological alterations were less obvious. Accordingly, the highest percentage of dead NTS, about 30%, was induced by RESV at higher concentration (Table 2). However, similar to what was observed above, following incubation in 10–100 μM PZQ for 1 h, the NTS were overactive and showed visible granularity while those incubated for 72 h showed severe damage, although the worms remained motile. Despite the severe damage induced by PZQ, about 50% of NTS remained alive (Table 2). Nonetheless, NTS incubated with the combined anthelmintic and RESV showed more morphological alterations at all concentrations, in terms of granularity, minimal movement, and alteration in shape, in comparison to NTS incubated with the same compounds alone (Additional file 1: Figure S4).

**Table 2:** Percentage (mean ± SD) of dead NTS induced by compounds alone and its combinations for different concentrations obtained by staining with iodide propidium

Concentration (μM)	Control	PZQ	AS	RESV	PZQ + RESV	AS + RESV
10	0.38 ± 0.18	45.7 ± 2.1	18.0 ± 3.5	12.3 ± 2.0	38.2 ± 3.7	23.7 ± 4.0
50	1.50 ± 0.71	52.7 ± 0.7	37.0 ± 3.4	28.1 ± 3.1	69.2 ± 2.8	35.5 ± 5.4
100	1.38 ± 0.88	56.9 ± 2.5	70.0 ± 3.8	30.0 ± 1.6	81.0 ± 5.2	99.9 ± 0.1

**Fig. 3:**
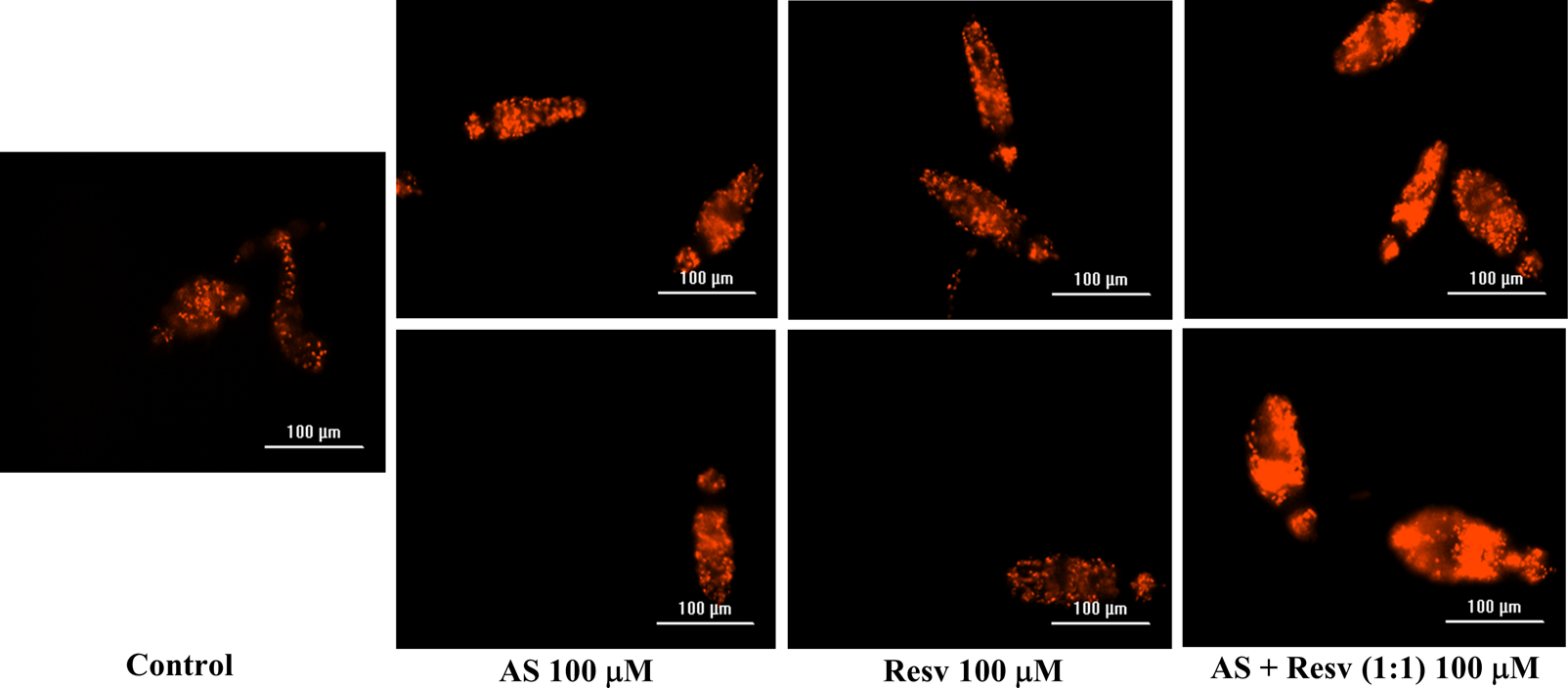
*In vitro* effects of AS, RESV and AS + RESV (highest concentration) on viability of *Schistosoma mansoni* NTS assessed by propidium iodide (PI) incorporation following exposure for 72 h. Notably, NTS exposed to AS + RESV showed stronger fluorescence than schistosomula cultured in AS or RESV alone, indicating that the former was dead. Images were captured using the BioTek LionHeart FX Automated Live Cell microscope (magnification of 20×) fitted with a 586 nm (Texas Red) filter. *Scale-bars*: 100 µm

**Fig. 4:**
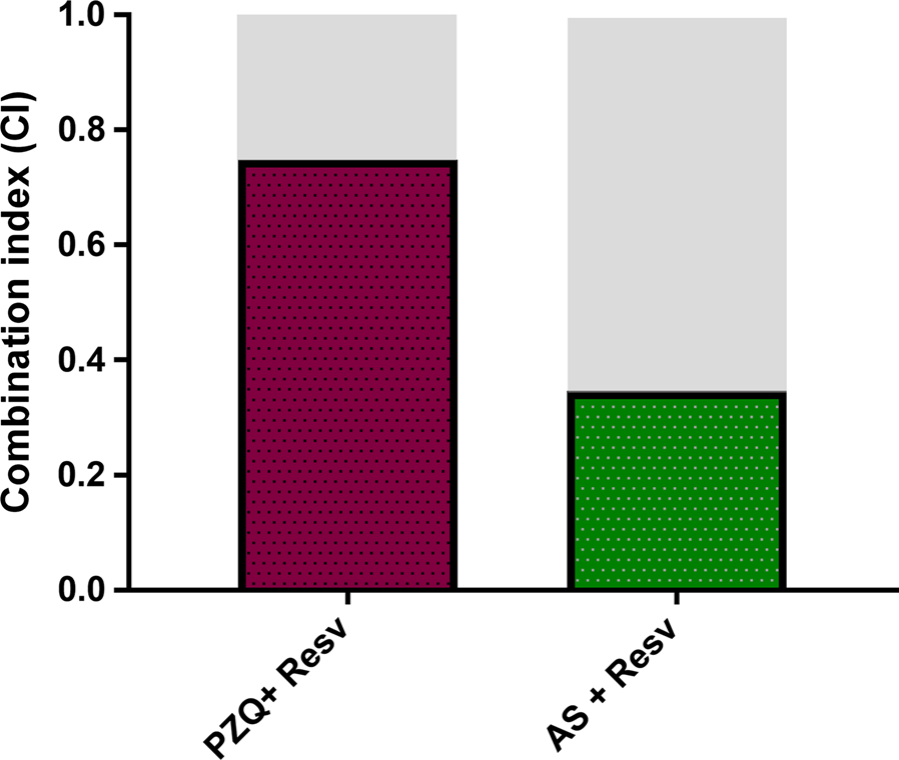
Combination indices (CI) obtained for combinations of anthelmintic drugs and RESV against newly transformed schistosomula of *S. mansoni*. The combination of AS + RESV was synergistically active against the schistosomula. High synergism is indicated in gray

In addition to the bright-field microscopical-based assessment, NTS viability was also assessed incorporating a red-fluorescent dye that objectively detects parasite survival during *in vitro* culture. Following 72 h, dead NTS were stained with PI and the plate examined was readout using the Texas Red filter at 586 nm on a BioTek LionHeart Automated Live Cell microscope (Fig. 3). As expected, all NTS incubated with AS-RESV were stained and were even brighter than others indicating that they were dead (Table 2). It is noteworthy that the number of dead schistosomula was lowest in control, followed by RESV, PZQ, AS and PZQ + RESV (Table 2). All combinations of anthelmintic drugs with RESV yielded synergistic antischistosomal effects. Based on these findings, we conclude that the combination of AS + RESV was identified as synergistic (CI = 0.34), near to strong synergism; and moderate synergism was observed for PZQ and RESV (CI = 0.74). These findings conformed with the microscopical observations: the combination of AS with RESV was more active against *S. mansoni* NTS *in vitro* than PZQ + RESV (Table 2, Fig. 4).

### Transmission electron micrographs revealed that combination induces significative internal damage

**Fig. 5:**
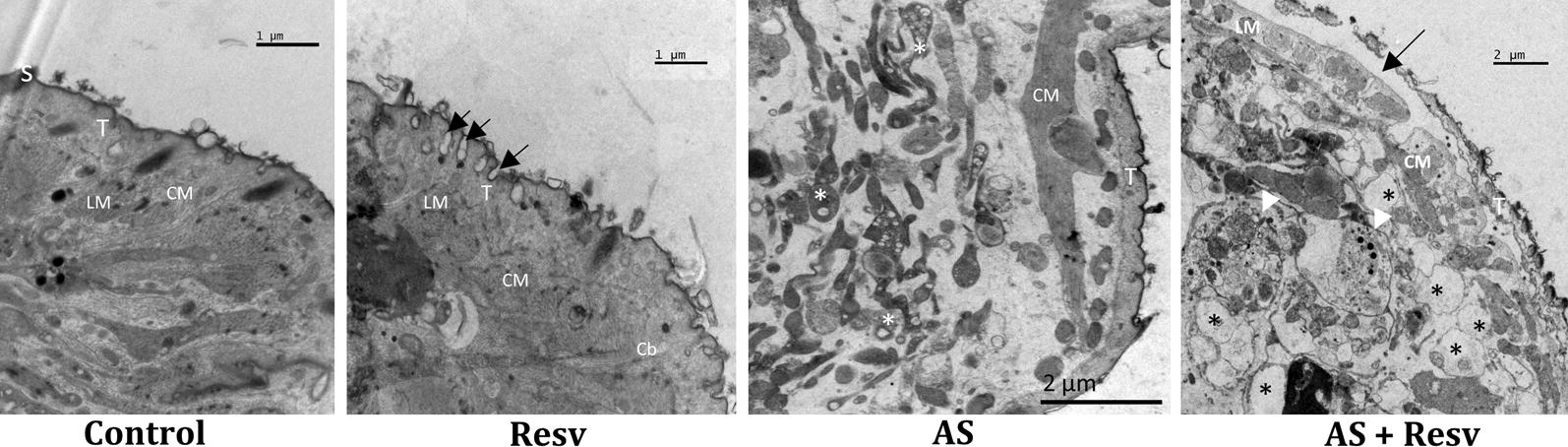
Ultrastructural level micrographs of schistosomula of *S. mansoni* at 72 h after exposure to AS, to RESV and to the combination of AS + RESV. In the control group, schistosomula exhibits intact tegument (T), spines (S), circular and longitudinal muscle (CM, LM) with a regular morphology. For NTS exposed to RESV, vesicles were seen in the tegument (arrows) along with some disorganization; no apparent damage occurred in subtegumental regions. In contrast, for schistosomula exposed to AS, loss of matrix integrity but without alterations to the tegument was apparent. The most prominent damage was seen on NTS treated with the combination of AS + RESV. Here, NTS displayed disruption and lysis of internal structures (arrowheads) and swelling of parenchyma tissues, disruption of tegument and disappearance of basal membrane (arrow), and the appearance of large vacuoles (asterisks). *Abbreviations*: T, tegument; S, spine; LM, longitudinal muscle; CM, circular muscle; Cb, cytoplasmatic bridge. *Scale-bars*: 1 µm (Control and Resv); 2 µm (AS and AS + Resv)

TEM analysis was employed to investigate and compare ultrastructural damages induced by AS, RESV alone or in combination (AS + RESV) (Fig. 5). The ultrastructural features of NTS in the control group remained normal and were similar to that in reported earlier [46]. The analysis of NTS treated with AS, RESV alone or combined (AS + RESV) at 100 μM revealed marked alteration of ultrastructural features of tegument and subtegumental structures (Fig. 5). Following exposure to RESV, vesicles were present on the tegument and there was irregularity in the appearance of the membrane. TEM evaluation of NTS treated with AS revealed the loss of matrix integrity diffusely through subtegumental regions and the presence of vacuoles on the interior of organelles, which probably resulted from cytoplasmic processes. Nonetheless, there was no evidence of significant alterations of the tegument. By contrast, for NTS treated with AS + RESV, tegumental disruption and disappearance of basal membrane were apparent. In addition, lysis of the tegumental matrix was usually revealed close to the basal membrane leading to the formation of large vacuoles above the basal membrane. The subsequent dysfunction or leakage of internal contents might be involved upon cytoplasmic lysis. It seems likely that disruption of the tegument might be directly linked to death of NTS treated with AS + RESV. Upon comparison of the ultrastructure of NTS treated with these compounds alone or in combination, it was possible observe that the damage was more prominent following combination treatment, reinforcing the notion that that RESV enhanced the antischistosomal activity of AS. The TEM micrographs were consistent with the findings obtained by light microscopy where it was possible to observe the presence of membrane disruption in the NTS treated with AS + RESV and extensive morphological alterations in contrast to the effects of AS or RESV alone (Fig. 2).

## Discussion

Schistosomiasis is a major public health and economic burden in the tropical developing world [6, 47, 48]. Currently, there is one drug available as the core treatment of schistosomiasis, and it has been used extensively in mass administration for transmission control [49]. Problematically, emergence of PZQ-resistance is not unlikely, and indeed low cure rates with PZQ have been repeatedly reported [10, 15]. There is a pressing need for new therapeutic approaches that combine different modes of action and/or repurposing of drugs [17]. Combination chemotherapy is common in medicine, including treatment for cancer, bacterial infections, HIV and malaria, as well as in the veterinary and agricultural arenas [50, 51].

Here, after establishing tractable transformation and culture conditions for NTS, drug sensitivity screenings were undertaken using bright field, visual inspection with an inverted microscope and also by automated microscopy using a BioTek LionHeart system. AS was more active than PZQ on NTS, which is consistent with earlier findings that showed that AS is more effective against larval and immature mammalian stages of *S. mansoni* due to the presence of the endoperoxide bridge that induces the production of ROS. By contrast, PZQ is more active against adult schistosomes [20]. Alone, NAC and RESV demonstrated modest activity against NTS. Although NAC did not exhibit antischistosomal activity against NTS *in vitro*, it might ameliorate redox homeostasis and host morbidity by downregulating oxidative stress caused by infection [26]. RESV induced more marked morphological changes than NAC on schistosomula; perhaps RESV acted on neuromotor activity, based on its effects on motility, which in turn could degrade its ability to migrate and acquire nutrients. Indeed, in *S. mansoni* infected mice, the administration of RESV ameliorates oxidative stress and organ dysfunction [30]. These effects likely are not only related to biological properties of RESV but also host antischistosomal activity. RESV might promote a combined action by both harming the schistosome while also ameliorating host oxidative stress.

With respect to the combinations, NAC did not enhance the activity of AS or PZQ. By contrast, AS or PZQ in combination with RESV improved performance in terms of antischistosomal effect, more than the single compound. This finding indicated that RESV possesses and enhances antischistosomal activity of both these anthelmintics. These outcomes were evident following serial dilution of AS, PZQ and RESV (alone) and combined. At dilute concentration, augmented activity against NTS was evident. Indeed, combinations of PZQ or AS with RESV presented a moderate (CI = 0.74) and marked (CI = 0.34) synergistic effect, respectively. Synergy in antischistosomal action might result from increasing the action on anthelmintic drugs targets or acting concomitantly on different targets [52].

The ultrastructural analysis demonstrated that NTS treated with AS + RESV suffered extensive and severe damage in comparison to controls and NTS treated with AS or RESV. The tegumental and subtegumental regions of these NTS showed alterations including disruption of tegument, extensive lysis of subtegumental regions with presence of numerous and vacuoles with diverse sizes, and loss of the basal membrane. Nonetheless, NTS treated with AS or PZQ alone also showed ultrastructural alterations, including loss of integrity of the matrix in the case of AS, and presence of vesicles on the tegument and some tegument disorder of larvae treated with RESV. With regard to controls, these presented regular morphology. Taken together, the light microscopic and TEM micrographs revealed that RESV not only induced alterations on the tegument of NTS but also augmented the antischistosomal activity of AS, leading to disruption of tegument and extensive lysis of subtegumental regions. The tegumental damage might lead might to disappearance of the immunological camouflage of the parasite which, in turn, would expose immunogens and immunogenic epitopes. These kinds of damage and immunological reactions represent a key process in the action of PZQ *in vivo* [53–56]. The schistosome tegument represents the frontline interface between host and parasite and plays a pivotal role in defence function to escape the host immune response. Additionally, it has essential secretory and nutrient absorption functions [57, 58]. Accordingly, tegumental disruption induced by AS + RESV would be anticipated to negatively affect the parasite’s capacity to support its nutrition and to thwart host immune responses.

The assessment of parasite viability microscopically *in vitro* is based on regular lack of movement of larvae (motility) and morphological changes such as granularity and shape alterations [37] that might be subjective and inaccurate [41]. Accordingly, it is crucial to complement microscopic examination with fluorometric or staining approaches. We used a simple method based on the incorporation of PI for red fluorescence staining that does not require an extensive knowledge of schistosome biology [40]. A good correlation was observed between light and fluorescence microscopic readouts after exposing schistosomula to anthelmintics and RESV (alone) and combined, indicating that microscopic readout complemented with fluorometric methods represented an accurate and tractable technique to assess viability. Other methods to objectively quantify the activity of antischistosomal drugs also are available, including the xCELLigence approach [59, 60].

RESV and AS exhibited antischistosomal activity against schistosomula and synergism of antischistosomal effect was seen RESV or AS was combined with AS or PZQ. This synergistic effect was most pronounced with AS + RESV. Based on these findings, a combination of active agents, preferably with discrete modes of action including activity against developmental stages and to ameliorate infection associated pathology, might be pursued in order to identify novel therapeutic interventions. Investigation also should be undertaken to assess the synergies of these combinations against adult forms, other schistosome species, and schistosome infections in laboratory rodents. Indeed, we intend to evaluate these combinations in the *S. haematobium*-hamster model [24, 61] and for related trematodes responsible for hepatobiliary tract disease including cholangiocarcinoma [24, 62–66].

## Conclusions

To conclude, RESV appeared to exhibit antischistosomal activity against schistosomula and also to induce synergism in combination with AS or PZQ. Based on these findings, we suggest that novel therapeutic interventions should be sought that involve the combination of active agents, preferably agents with discrete modes of action, and which also exhibit activity against developmental stages, and/or which also ameliorate infection-associated pathology.

## Additional file

**Additional file 1: Figure S1.** Morphology of *S. mansoni* NTS after 96 h incubation using different supplemented M199. **Figure S2.** Viability score induced by drugs, antioxidants alone or combined. **Figure S3.** Morphological alterations induced by drugs (PZQ and AS), antioxidants (Resv and NAC) alone or combined. **Figure S4.** Morphological alterations observed at different concentrations of compounds. **Figure S5.** Graphical of percentage of dead NTS induced by compounds evaluated at different concentrations. **Table S1.** Percentage of dead NTS induced by compounds alone and combination evaluated at 100 μM and constant ratio 1:1.

## Data Availability

All data generated or analysed during this study are included in this published article and its additional file.

## Abbreviations

AS: artesunate
CI: combination index
DMSO: dimethylsulfoxide
iFBS: heat inactivated fetal bovine serum
NAC: N-acetylcysteine
NTS: newly transformed schistosomula
PI: propidium iodide
PZQ: praziquantel
RESV: resveratrol
TEM: transmission electron microscopy
