# Supplementary material for: The antioxidants resveratrol and N-acetylcysteine enhance anthelmintic activity of praziquantel and artesunate against Schistosoma mansoni
Source: Parasit Vectors. 2019 Jun 20;12:309. doi: 10.1186/s13071-019-3566-9 (PMC6585032; doi:10.1186/s13071-019-3566-9)

# Additional File 1

**Figure S1 -** Morphology of *S. mansoni* NTS after 96h incubation using different supplemented M199.

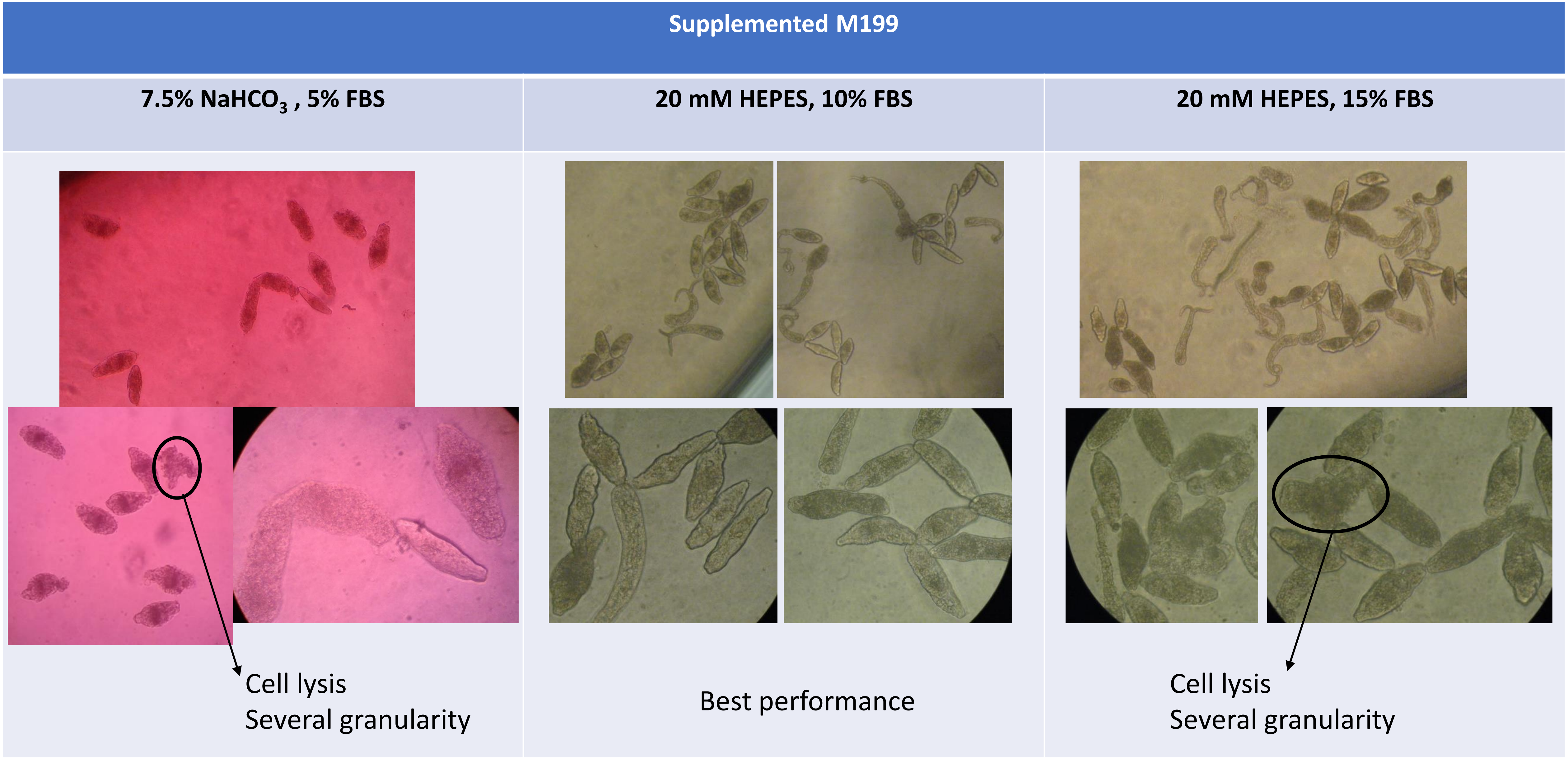

**Figure S2 -** Viability score induced by drugs, antioxidants alone or combined.

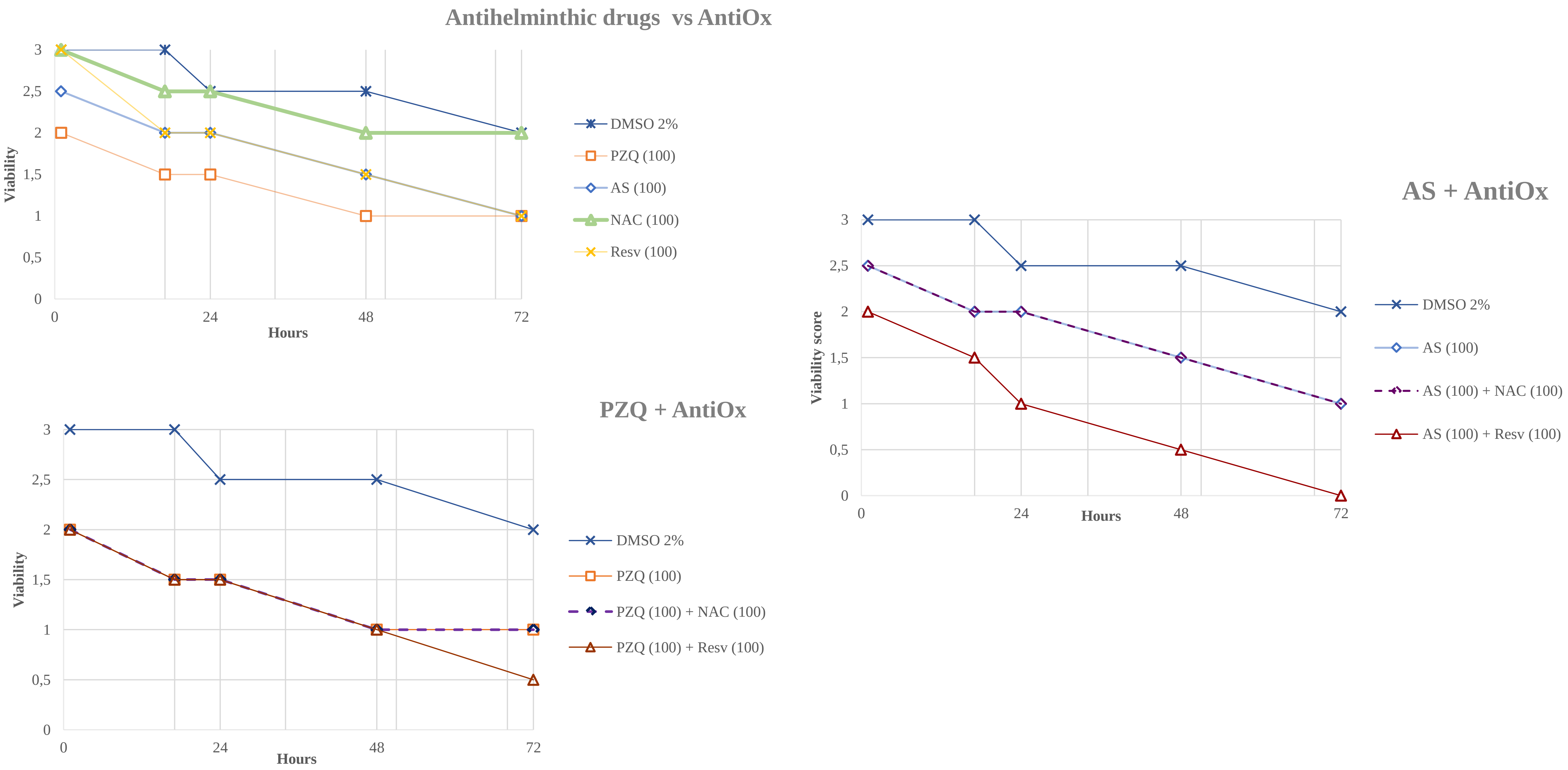

**Figure S3 –** Morphological alterations induced by drugs (PZQ and AS), antioxidants (Resv and NAC) alone or combined.

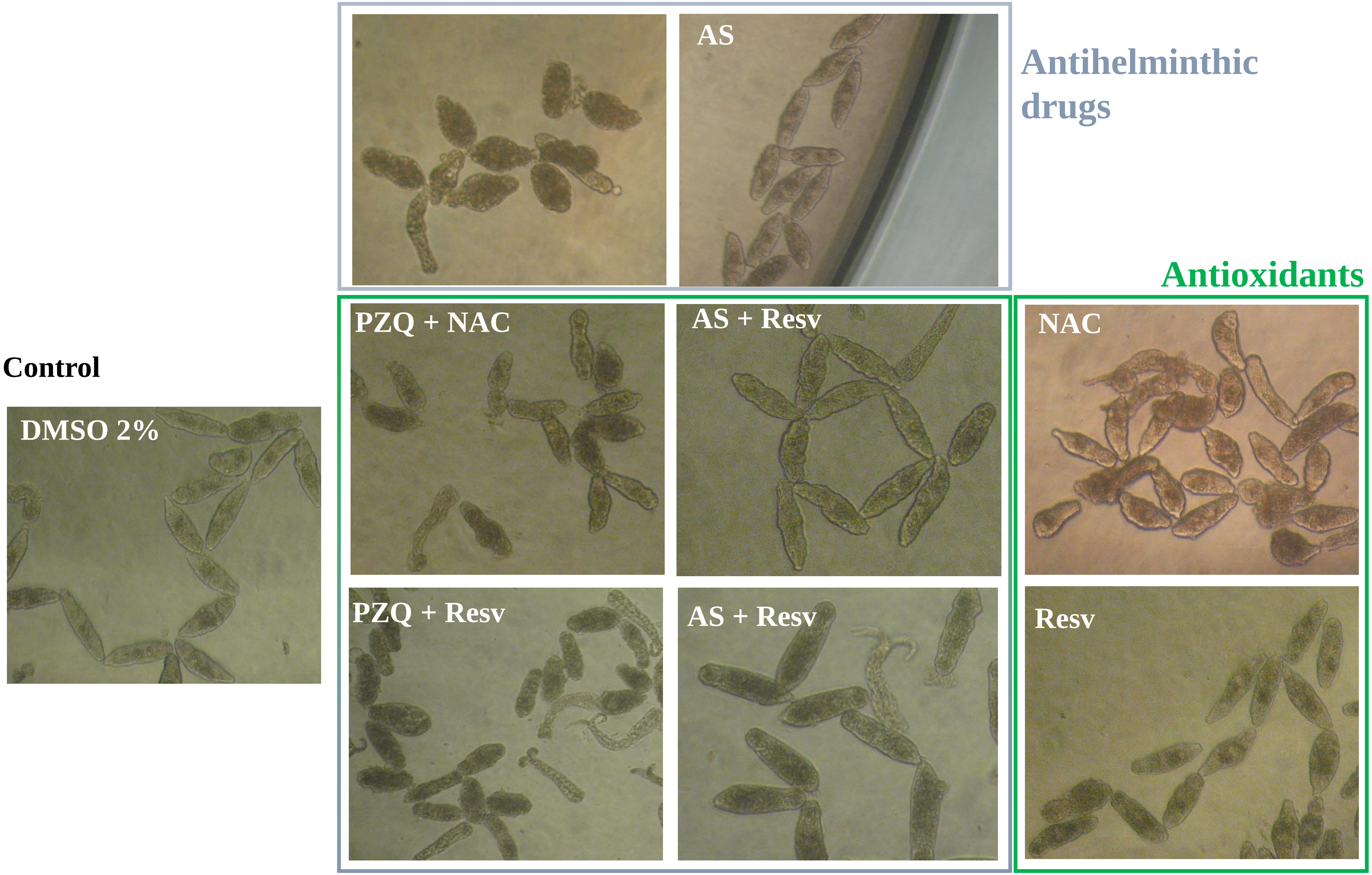

**Figure S4** - Morphological alterations observed at different concentrations of compounds.

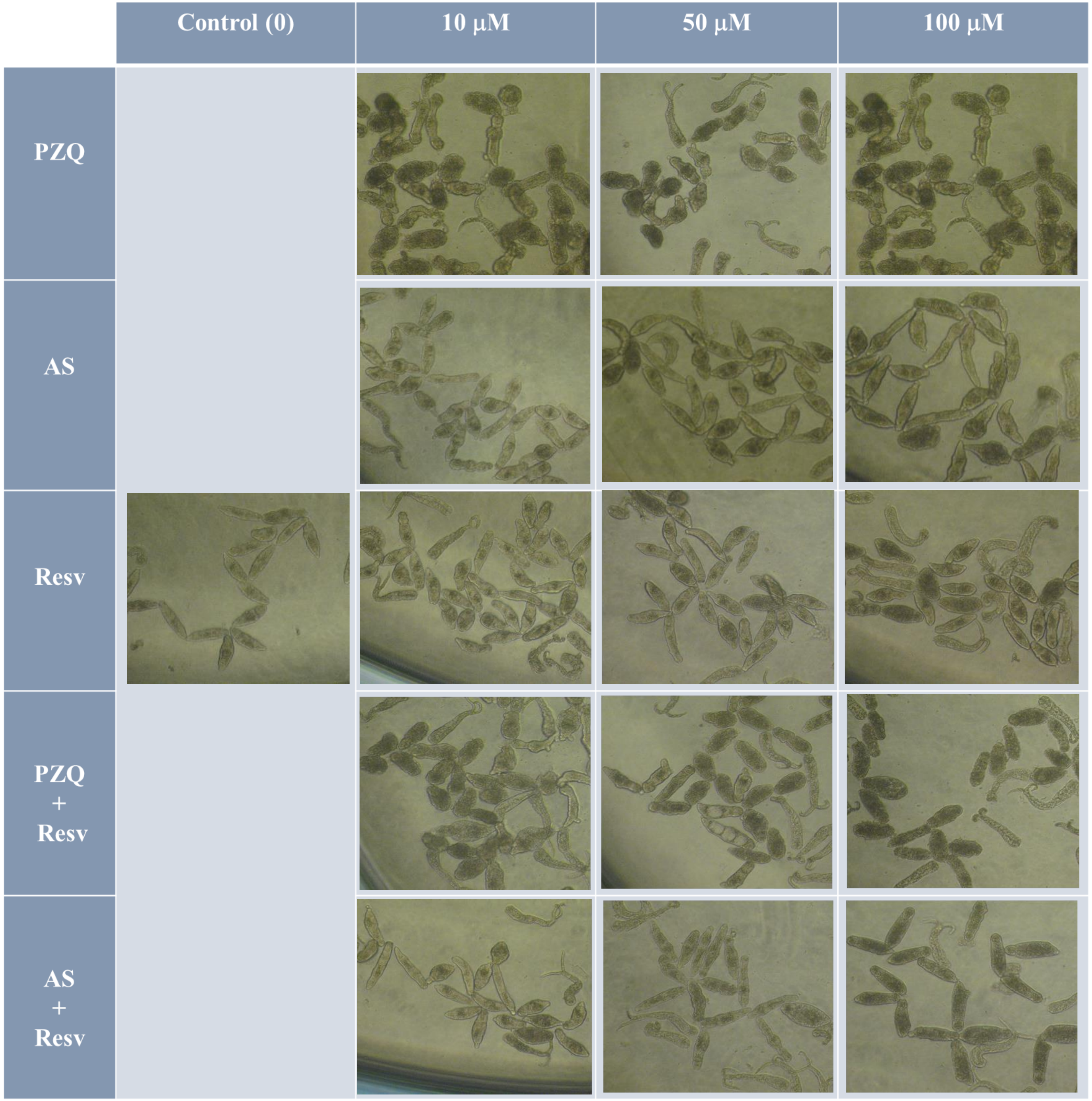

**Figure S5 –** Graphical of percentage of dead NTS induced by compounds evaluated at different concentrations.

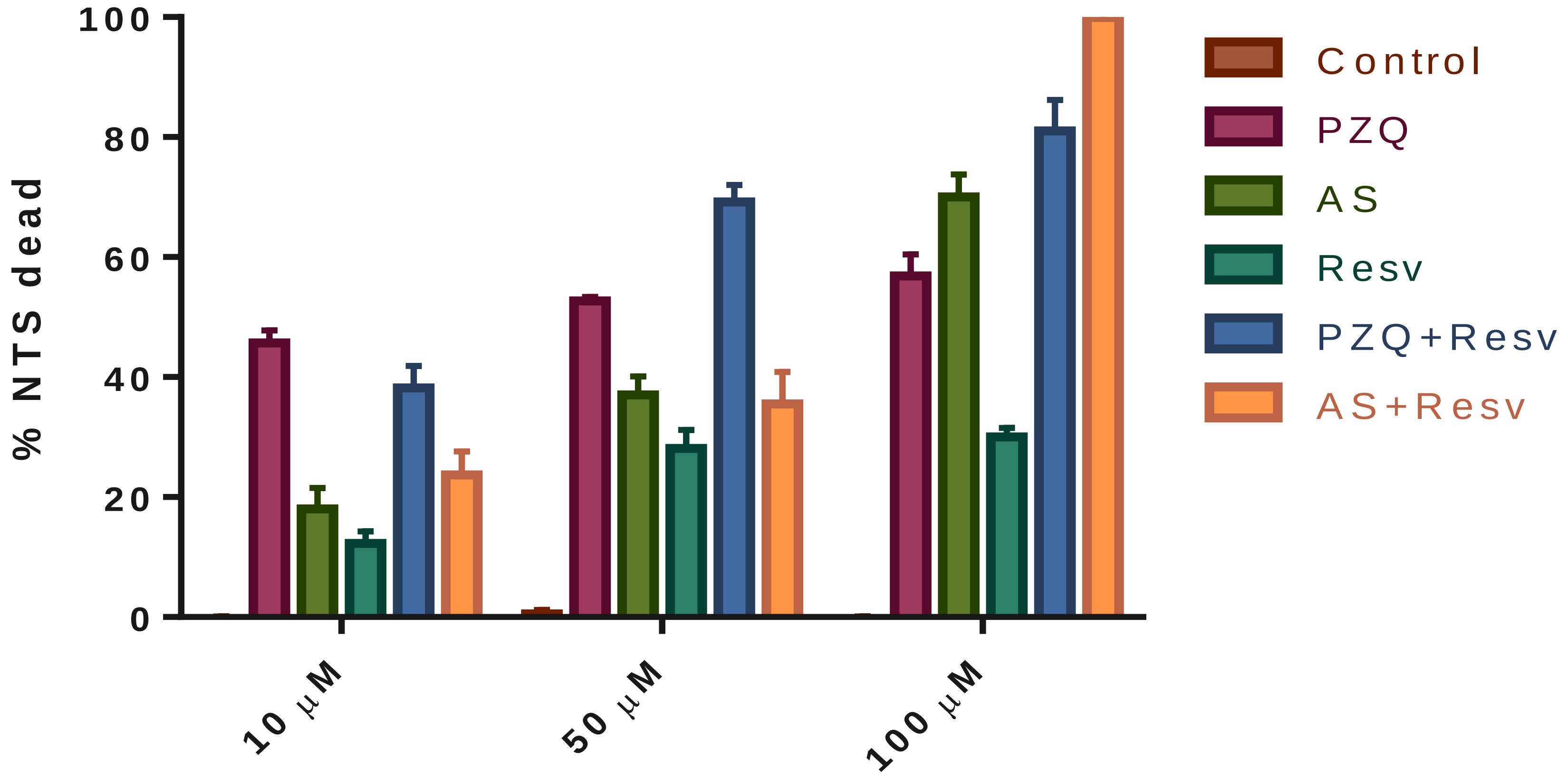

**Table S1:** Percentage of dead NTS induced by compounds alone and combination evaluated at 100 μM and constant ratio 1:1. (N.D., not determinate)

| | Mean ± SD | | | | | | | | |
|---|---|---|---|---|---|---|---|---|---|
| ***[conc. μM]*** | **Control** | **PZQ** | **AS** | **RESV** | **NAC** | **PZQ+RESV** | **PZQ+NAC** | **AS+RESV** | **AS+NAC** |
| **100** | 1.38±0.88 | 56.9±2.5 | 70.0±3.8 | 30.0±1.6 | 7.8±N.D. | 81.0±5.2 | 59.35±26.4 | 99.9±0.1 | 15.7±N.D. |

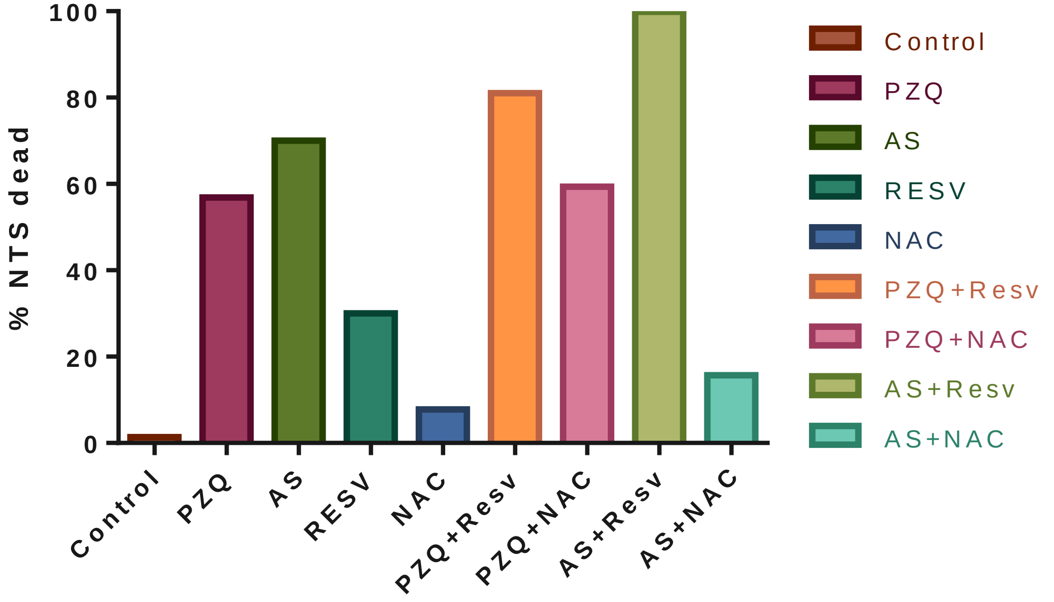

Supplement: Supplementary file 1 — Additional file 1: Figure S1. Morphology of S. mansoni NTS after 96 h incubation using different supplemented M199. Figure S2. Viability score induced by drugs, antioxidants alone or combined. Figure S3. Morphological alterations induced by drugs (PZQ and AS), antioxidants (Resv and NAC) alone or combined. Figure S4. Morphological alterations observed at different concentrations of compounds. Figure S5. Graphical of percentage of dead NTS induced by compounds evaluated at different concentrations. Table S1. Percentage of dead NTS induced by compounds alone and combination evaluated at 100 μM and constant ratio 1:1. [file 13071_2019_3566_MOESM1_ESM.pdf]
